# Rural versus urban variations of factors associated with early initiation of breastfeeding in Ethiopia

**DOI:** 10.1016/j.heliyon.2024.e33427

**Published:** 2024-06-23

**Authors:** Desalegn Girma, Zinie Abita

**Affiliations:** a Department of Midwifery, College of Health Science, Mizan-Tepi University, Mizan Teferi, Ethiopia; b School of Public Health, College of Health Science, Mizan-Tepi University, Mizan Teferi, Ethiopia

**Keywords:** Breastfeeding, Early initiation, Factors, Rural-urban, Ethiopia

## Abstract

**Background:**

Early initiation of breastfeeding is an important strategy to reduce under-five mortality. Nevertheless, it remains under-practiced in developing countries. In Ethiopia, there were studies done to identify determinants of early initiation of breastfeeding. However, the variation of factors among rural versus urban residents has not been investigated. Therefore, the main objective of this study is to investigate the variation of factors associated with early initiation of breastfeeding among rural versus urban residences.

**Methods:**

The 2016 Ethiopian Demographic and Health Survey data was used to conduct the study. Mothers whose index child aged less than 24 months have participated in the study. We excluded mothers who had fetal death during birth and who didn't live with their child. Accordingly, a total of 3396 weighted samples of mothers from rural residences and 478 weighted samples of mothers from urban residences were included in the final analysis. A multivariable logistic regression analysis has been used to explore determinants of early initiation of breastfeeding. Finally, statistically significant associations have been declared by using AOR with a 95%CI at a p-value of <0.05.

**Results:**

In rural residences, age of mothers 15–24 years (AOR: 1.39, 95 % CI: 1.08, 1.79), mothers who are not currently working (AOR: 1.45, 95 % CI: 1.19, 1.78), large birth size (AOR: 1.49, 95 % CI: 1.17, 1.92), and giving birth at health facility (AOR: 1.25, 95 % CI: 1.01, 1.53) were factors associated with a higher odds of early initiation of breastfeeding. Whereas, in urban residences, being second to third birth order (AOR: 1.94, 95 % CI: 1.01, 3.75), skin-to-skin contact care (AOR: 2.58, 95 % CI: 1.44, 4.63) and antenatal care visit were factors associated with early initiation of breastfeeding. Regardless of residences, vaginal delivery (Rural AOR: 4.06, 95 % CI: 1.75, 9.44; Urban AOR: 2.52, 95 % CI: 1.15, 5.54) and involvement of mothers in health care decisions (Rural AOR: 1.52, 95 % CI: 1.25, 1.85; Urban AOR: 2.62, 95 % CI: 1.33, 5.17) were common determinants of early initiation of breastfeeding.

**Conclusions:**

This study concludes that the factors associated with early initiation of breastfeeding are different among rural versus urban residences. Accordingly, maternal ages, maternal current working status, birth sizes, and place of delivery are identified as factors associated with early initiation of breastfeeding among rural residences. Whereas, antenatal care visits, skin-to-skin contact care, and birth order are identified as factors associated with early initiation of breastfeeding among urban residences. Regardless of residence, mode of delivery, and involvement of mothers in health care decisions are common determinants of early initiation of breastfeeding. Therefore, irrespective of the residence, special emphasis has to be given to newborns delivered by cesarean section to increase the rate of early initiation of breastfeeding.

## Highlights

• Starting breastfeeding immediately after birth is important to prevent newborn deaths during the riskiest time of life.
• There was a significant variation of factors associated with early initiation of breastfeeding among rural and urban residents.
• Cesarean section delivery was negatively associated with early initiation of breastfeeding, regardless of the residence of mothers.
• Strategies should be endorsed against the identified factors respective to the residence of mothers (rural versus urban).

## 1 Introduction

Worldwide, an estimated 2.4 million newborn deaths were reported in 2019 [1]. Early initiation of breastfeeding is one of the recommended strategies to reduce neonatal mortality [[2], [3], [4]]. Early initiation of breastfeeding is commencing of breastfeeding within the first hour after birth [3]. It is a baseline for the success of exclusive breastfeeding among infants [5,6].

Early initiation of breastfeeding assures the mothers that the newborn receives colostrum (initial milk), which contains nutritionally important antibodies and nutrients [7]. Hence, It shields newborns from passing during the most hazardous time of life [2]. Early initiation of breastfeeding can decrease the rate of neonatal mortality by 45 percent while exclusive breastfeeding increases the survival rate of infants by 14 times [8]. Previous evidence affirmed that appropriate breastfeeding prevents the death of 823,000 children each year [9]. It also important for the health of mothers as it avoids postpartum hemorrhage, enhances mother-to-child bonding [10], and prevents ovarian [11] and breast cancer [9].

Although early initiation of breastfeeding has a tremendous benefit, globally, only 46 % of newborns started breastfeeding within 1 h after birth [12]. A study conducted in 57 low and middle-income countries revealed that only 51.9 % of newborns started breastfeeding within 1 h after birth [13]. Similarly, in Sub-Saharan African countries, only 58.3 % of newborns were placed to breast within 1 h with significant disparity among nations, ranging from the lowest in Chad (24 %) to the highest in Burundi (86 %) [14]. In Ethiopia, though there has been an improvement in early initiation of breastfeeding from 48.8 % in 2000 to 75.7 % in 2016 [15], the proportion is still far from the national strategic target of 92 percent by the end of 2015 [16]. Here, the implication is that there are factors that need further study.

In Ethiopia, studies have been done so far to assess determinants of early initiation of breastfeeding among newborns. So, maternal educational status [[17], [18], [19], [20]], mode of delivery [17,18,[21], [22], [23]], parity [18,21], place of delivery [15,18,22,24,25], residence [[18], [19], [20],^26^], income [18,27], antenatal care visit (ANC) [15,18,23], number of family members [15], maternal working status [20], child sex [20], number of children [19], birth attendant [23], husband education [27] and knowledge about child feeding [27] were determinants of early initiation of breastfeeding. However, these studies have been done in corner parts of Ethiopia and can't represent the nation at large. Although there has been one study done at the national level [28], it reports the aggregated result of early initiation of breastfeeding among all populations, and yet, segregation by rural versus urban populations has not been done. In other ways, the variation of factors among rural versus urban populations has not been investigated. Such that, using the aggregated finding may cover the difference of factors among rural versus urban residences. Thus, there is a gap in how the determinants of early initiation of breastfeeding differ across rural versus urban residences. Hence, we follow the rural versus urban stratification method to unveil equity gaps among socioeconomic classes or geographic residences. Moreover, World Health Organization (WHO) recommends further research among regions, countries, and population groups to enhance optimal breastfeeding practices [3]. Therefore, the main objective of this study is to identify the determinants of early initiation of breastfeeding among rural versus urban residence using nationally representative data. The finding of this study could help the policymakers or researchers to endorse strategies and programs related to breastfeeding by identifying its determinants along with the respective residences.

## 2 Methods

### 2.1 Study area and data source

**Fig. 1:**
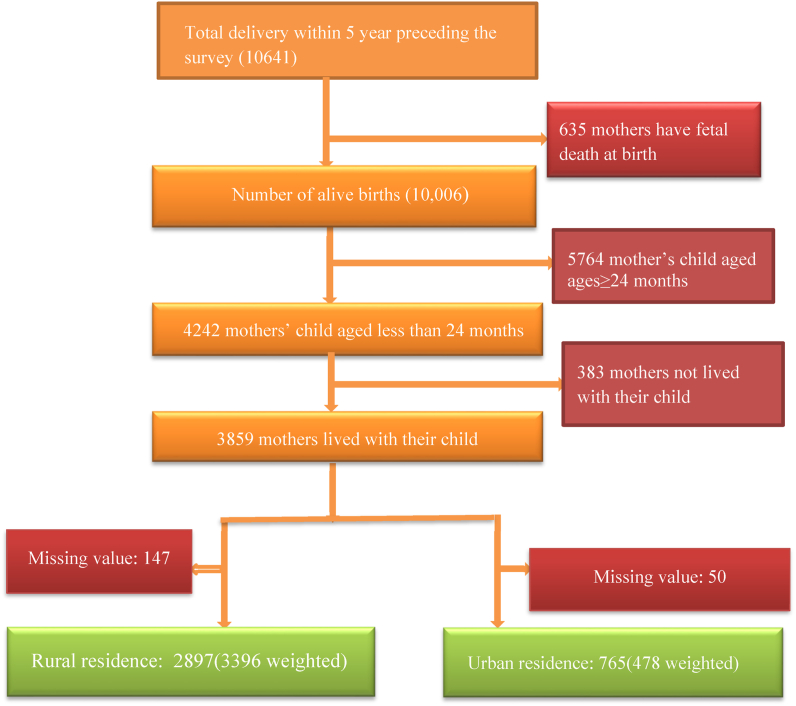
schematic presentation of study populations.

The 2016 Ethiopian Demographic and Health Survey data was used to conduct the study. It was a national level Survey conducted from January 18 to June 27, 2016. A cross-sectional study design was used. A two-step stratified sampling procedure was employed. A total of six hundred forty-five enumeration areas, comprising 443 rural and 202 urban areas, were chosen at the first stage. Then, 28 homes have been chosen from each cluster in the second phase. Moreover, the full description of the sampling procedure was outlined in the EDHS 2016 reports [29]. A total of 10,641 women who gave birth before the five-year survey drawn from the births data set were used as a source population. We included all mothers whose index child (aged less than 24 months). Whereas, we excluded mothers who had fetal death during birth and mothers who didn't live with their child. Accordingly, a total of 3396 weighted samples of mothers from rural residences and 478 weighted samples of mothers from urban residences have been used for our analysis (Fig. 1).

### 2.2 Variables

Early initiation of breastfeeding is defined as the initiation of breastfeeding for the newborn within 1 h of birth [30]. Mothers have been asked when they started breastfeeding their baby after birth and the responses have been categorized as “early initiated” (initiated within the first hour, coded as “1”) and late initiated (initiated after 1 h, coded as “0”). Hence, the outcome variable is represented as $Y_{ij}$ = $\left\{ \begin{array}{c} Early\;initiation\;of\;bereastfeeding\\ Late\;initiation\;of\;bereastfeeding \end{array}\right.$, the category is dichotomous.

Age of mothers, education status of mothers, current working status of the mother, husband education, number of under-five children, wealth index, media exposure, first childbearing age, child age, place of childbirth, parity, women involvement in health care decisions, ANC, mode of delivery, child sex, birth type, skin to skin contact care (SCC), birth size, birth order, and regions [small peripheral regions (Afar, Somali, Benishangul Gumz, and Gambela), large central regions (Tigray, Amhara, Oromia, Harari, and South Nations Nationalities and Peoples Region (SNNPR)), and metropolitans (Dire Dawa, and Addis Ababa] were independent variables included in this study **(**Supplementary Table 1**).**

### 2.3 Statistical analysis

Data was summarized using frequencies and percentages. Data weighting was done for sample probabilities and non-response using the factor supplied in the EDHS data to keep the representativeness of the survey and get reliable estimates. A chi-square test was done to compare the rate of early initiation of breastfeeding according to the study characteristics. Bivariate logistic regression analysis has been used to identify the crude association of each independent factor with the dependent variable. In bivariate logistic regression analysis, factors that have an association with early initiation of breastfeeding at a p-value of <0.25 were candidates for multivariable logistic regression analysis. Finally, a multivariable logistic regression model was fitted to identify the adjusted association of factors with early initiation of breastfeeding and statistically significant associations were declared at a p-value of <0.05. The Hosmer–Lemeshow test (i.e. P-value >0.05) was done to determine the model goodness of fit. All the above analyses were conducted separately for data segregated by rural versus urban residences.

### 2.4 Ethics approval and consent to participate

Participant consent and ethical approval were not necessary in this case because we used secondary data from the MEASURE DHS program that was made publicly available. Permission to download the data from (https://dhsprogram.com/data/dataset_admin/login_main.cfm) was granted after we requested the DHS Program. The requested data were solely used for research and were used anonymously. The EDHS-2016 report contained all relevant information regarding the ethical issue.

## 3 Results

### 3.1 The rates of early initiation of breastfeeding among rural versus urban residence

The rate of early initiation of breastfeeding among rural residences was 71.8 % (95 % CI: 70.2, 73.4); whereas among urban residences was 77.9 % (95 % CI: 75.0, 80.7). In rural residences, the rate of early initiation of breastfeeding was lower among newborns whose mothers are currently working (22.2 %) than newborns whose mothers are not currently working (77.8 %). Similarly, in rural residences, the rate of early initiation of breastfeeding was lower among newborns whose birth sizes are small (27 %) and large (30 %) than newborns whose birth sizes are average (43 %) Whereas, in urban residences, the rate of early initiation of breastfeeding was significantly lower among newborns whose mothers have primary educational (33.8 %)and didn't have formal educational (23.9 %) than newborns whose mothers have secondary and above educational level (42.2 %). Moreover, the rate of early initiation of breastfeeding was higher among newborns who received SCC (67.6 %) than newborns who didn't receive SCC (32.4 %). Furthermore, in urban residences, there was also a high rate of early initiation of breastfeeding among newborns whose mothers have ≥4 ANC (66.8 %) than newborns whose mothers didn't have ANC (5.1 %) (Table 1).

**Table 1:** Sample characteristics and rates of early initiation of breastfeeding by rural versus urban residences in Ethiopia, 2016.

Variables	Categories	Rural					Urban				
		Unweighted N (%)	Weighted N (%)	Unweighted rate of EIBF N (%)	Weighted Rate of EIBF N (%)	P value	Unweighted N (%)	Weighted N (%)	Unweighted rate of EIBF N (%)	Weighted rate of EIBF N (%)	P value
**Maternal age (years)**	15–24	882 (30.4)	980 (28.9)	704 (31)	812 (29.3)	0.07	196 (25.6)	108 (22.5)	165 (25.3)	79 (20.1)	0.36
	25–34	1388 (47.9)	1704 (50.2)	1096 (48.3)	1413 (51.0)		455 (59.5)	291 (61)	391 (60.0)	246 (62.6)	
	≥35	627 (21.6)	712 (21.0)	471 (20.7)	543 (19.6)		114 (14.9)	79 (16.5)	96 (14.7)	68 (17.3)	
**Child age (month)**	<6	963 (33.2)	1120 (32.9)	714 (32.6)	876 (32.4)	0.13	246 (32.0)	159 (33.2)	193 (31.7)	125 (33.7)	0.74
	6–11	638 (20.5)	736 (20.4)	447 (20.4)	551 (20.4)		178 (21.9)	113 (22.3)	130 (21.4)	77 (20.8)	
	12–23	1296 (46.3)	1540 (46.7)	1031 (47.0)	1276 (47.2)		341 (46.1)	206 (44.6)	285 (46.9)	169 (45.6)	
**Maternal education status**	No formal education	1936 (66.8)	2215 (65.2)	1519 (66.9)	1806 (65.2)	0.72	189 (24.7)	116 (24.3)	167 (25.6)	94 (23.9)	0.02
	Primary	801 (27.6)	1050 (30.9)	623 (27.4)	857(31.0)		249(32.5)	151(31.6)	215(33.0)	133(33.8)	
	Secondary and above	160(5.5)	131(3.8)	129(5.7)	105(3.8)		327(42.7)	211(44.1)	270(41.4)	166(42.2)	
**Mother Currently working**	No	2187(75.5)	2614(77.0)	1748(77)	2153(77.8)	0.02	463(60.5)	261(54.7)	406(62.3)	217(55.4)	0.20
	Yes	710(24.5)	782(23.0)	523(23)	615(22.2)		302(39.5)	216(45.3)	246(37.7)	175(44.6)	
**Partner education status**	No formal education	1416(51.5)	1553(48.2)	1109(51.2)	1267(47.8)	0.56	101(14.6)	62(14.2)	90(15.1)	52(14.2)	0.49
	Primary	986(35.8)	1553(48.2)	787(36.3)	1133(42.8)		211(30.4)	144(32.9)	180(30.2)	124(34.0)	
	Secondary and above	350(12.7)	305(9.5)	271(12.5)	248(9.4)		382(55.0)	232(53)	326(54.7)	189(51.8)	
**Number of under-five children**	≤2	1062(36.7)	1249(36.8)	761(33.5)	920(33.2)	0.01	444(58.0)	306(64.2)	364(55.8)	242(61.7)	0.02
	>2	1835(63.3)	2145(63.2)	1510(66.5)	1847(66.8)		321(42.0)	171(35.8)	288(44.2)	150(38.3)	
**Wealth index**	Poor	1368(60.2)	1695(49.9)	1770(61.1)	1398(50.5)	0.13	50(6.5)	37(7.8)	42(6.4)	28(7.1)	0.12
	Medium	417(18.4)	808(23.8)	521(18.0)	662(23.9)		11(1.4)	4(0.8)	8(1.2)	1(0.3)	
	Rich	486(21.4)	893(26.3)	606(20.9)	708(25.6)		704(92.0)	436(91.4)	602(92.3)	363(92.6)	
**Exposed to media**	No	2185(75.4)	2424(71.4)	1717(75.6)	1987(71.8)	0.27	158(20.7)	92(19.2)	134(20.6)	72(18.3)	0.28
	Yes	712(24.6)	972(28.6)	554(24.4)	781(28.2)		607(79.3)	386(80.8)	518(79.4)	321(81.7)	
**Region**	Large to center	1574(54.3)	3186(93.8)	1229(54.1)	2594(93.7)	0.27	184(24.1)	329(68.8)	149(22.9)	270(68.7)	0.25
	small peripheral	1076(37.1)	3186(93.8)	803(35.4)	159(5.7)		195(25.5)	35(7.3)	176(27.0)	33(8.40	
	Metropolitan	247(8.5)	15(0.4)	239(10.5)	15(0.5)		386(50.5)	114(23.8)	327(50.2)	90(22.9)	

Variables	Categories	Rural					Urban				
		Unweighted N (%)	Weighted N (%)	Unweighted rate of EIBF N (%)	Weighted Rate of EIBF N (%)	P value	Unweight N (%)	Weighted N (%)	Unweighted rate of EIBF N (%)	Weighted rate of EIBF N (%)	P value
Age at first birth	<18years	1774(61.2)	1379(40.6)	1391(61.3)	1131(40.9)	0.27	165(21.6)	107(22.4)	146(22.4)	96(24.5)	0.28
	≥18 years	1123(38.8)	2016(59.4)	880(38.7)	1636(59.1)		600(78.4)	371(77.6)	506(77.6)	296(75.5)	
Place of delivery	Health facility	932(32.2)	1054(31.0)	748(32.9)	861(31.1)	0.21	670(87.6)	421(88.1)	573(87.9)	354(90.1)	0.004
	Home	1965(67.8)	2342(69.0)	1523(67.1)	1907(68.9)		95(12.4)	57(11.9)	79(12.1)	39(9.9)	
Parity	1–3	1362(47.0)	1611(47.5)	1066(46.9)	1301(47.0)	0.49	580(75.8)	369(77.2)	484(74.2)	299(76.1)	0.63
	4–5	685(23.6)	768(22.6)	531(23.4)	635(22.9)		121(15.8)	75(15.7)	110(16.9)	61(15.5)	
	≥6	850(29.3)	1016(29.9)	674(29.7)	832(30.1)		64(8.4)	34(7.1)	58(8.9)	33(8.4)	
Women participating in making health care decisions	Yes	2154(74.4)	2567(75.6)	1735(76.4)	2136(77.2)	0.01	644(84.2)	414(86.6)	552(84.7)	346(88.3)	0.02
	No	743(25.6)	829(24.4)	536(23.6)	632(22.8)		121(15.8)	64(13.4)	100(15.3)	46(11.7)	
Antenatal care visits	Not all	1111(38.4)	1307(38.5)	879(38.7)	1099(39.7)	0.67	46(6.0)	33(6.9)	35(5.4)	20(5.1)	0.03
	1-3 visits	912(31.5)	1095(32.3)	715(31.5)	868(31.4)		196(25.6)	128(26.8)	174(26.7)	110(28.1)	
	≥4 visits	874(30.2)	993(29.2)	677(29.8)	800(28.9)		523(68.4)	316(66.2)	443(67.9)	262(66.8)	
Mode of delivery	C/S	24(0.8)	26(0.8)	12(0.5)	14(0.5)	0.01	82(10.7)	45(9.4)	54(8.3)	31(7.9)	0.014
	vaginal	2873(99.2)	3369(99.2)	2259(99.5)	2753(99.5)		683(89.3)	432(90.6)	598(91.7)	361(92.1)	
Sex of child	Male	1430(49.4)	1622(47.8)	1103(48.6)	1294(46.8)	0.01	387(50.6)	251(52.4)	319(48.9)	200(50.9)	0.27
	Female	1467(50.6)	1773(52.2)	1168(51.4)	1473(53.2)		378(49.4)	228(47.6)	333(51.1)	193(49.1)	
Birth type	Single	2868(99.0)	3366(99.1)	2250(99.1)	2745(99.2)	0.43	757(99.0)	472(98.5)	645(98.9)	387(98.5)	0.85
	Multiple	29(1.0)	30(0.9)	21(0.9)	23(0.8)		8(1.0)	7(1.5)	7(1.1)	6(1.5)	
Skin-to-skin care	No	2132(73.6)	2515(74.1)	1659(73.1)	2048(74.0)	0.44	258(33.7)	176(36.8)	208(31.9)	127(32.4)	0.01
	Yes	765(26.4)	881(25.9)	612(26.9)	720(26.0)		507(66.3)	302(63.2)	444(68.1)	265(67.6)	
Birth size	Large	791(27.3)	1006(29.6)	650(28.6)	831(30.0)	0.01	247(32.3)	152(31.8)	215(33.0)	133(33.8)	0.12
	Average	1280(44.2)	1471(43.3)	996(43.9)	1189(43.0)		368(48.1)	223(46.7)	308(47.2)	178(45.3)	
	Small	826(28.5)	919(27.1)	625(27.5)	748(27.0)		150(19.6)	103(21.5)	129(19.8)	82(20.9)	
Birth Order	1	500(17.3)	590(17.4)	390(17.2)	475(17.2)	0.57	251(32.8)	162(33.9)	201(30.8)	119(30.3)	0.02
	2–3	862(29.8)	1020(30.0)	676(29.8)	826(29.8)		329(43.0)	207(43.3)	283(43.4)	180(45.8)	
	≥4	1535(53.0)	1785(52.6)	1205(53.1)	1467(53.0)		185(24.2)	109(22.8)	168(25.8)	94(23.9)	

### 3.2 Factors associated with early initiation of breastfeeding among rural residences

In bivariate logistic regression analysis, factors such as age of mothers, mother's current working status, wealth index, number of under-five children, place of childbirth, mode of delivery, child sex, size of the baby, and involvement of mothers in making health care decisions were the factors associated with early initiation of breastfeeding at a p-value of <0.25. Finally, in the multivariable logistic regression model, the ages of mothers, current working status of mothers, number of under-five children, mode of delivery, involvement of mothers in making health care decisions, and size of the baby were variables persistently associated with the outcome variable. Accordingly, the likelihood of early initiation of breastfeeding was increased by 39 % (AOR: 1.39, 95 % CI: 1.08, 1.79) among newborns whose mothers ages are between 15 and 24 years than newborns whose mothers ages are ≥35 years. The likelihood of early initiation of breastfeeding was increased by 45 % (AOR: 1.45, 95 % CI: 1.19, 1.78) among newborns whose mothers are not currently working than newborns whose mothers are currently working. In this study, mothers who have two or fewer children in the household were associated with lower odds of early initiation of breastfeeding (AOR: 0.52, 95 % CI: 0.43, 0.63) as compared to mothers who have two or more children. The likelihood of early initiation of breastfeeding was 1.52 times (AOR: 1.52, 95 % CI: 1.25, 1.85) higher among newborns whose mothers participated in making healthcare decisions than newborns whose mothers didn't participate in making healthcare decisions. The odds of early initiation of breastfeeding were 1.49 times (AOR: 1.49, 95 % CI: (1.17, 1.92) higher among newborns who have large birth sizes than newborns who have small birth sizes. In this study**,** newborns who had vaginal delivery were four times (AOR: 4.06, 95 % CI: 1.75, 9.44) more likely to start breastfeeding within the first hour than newborns who are delivered through cesarean section. The likelihood of early initiation of breastfeeding was increased by 25 % (AOR: 1.25, 95 % CI: 1.01, 1.53**)** among newborns who were delivered at a health institution than newborns who were delivered at home (Table 2).

**Table 2:** Shows the bivariate and multivariable logistics regression analysis of factors associated with early initiation of breastfeeding in rural residence, Ethiopia, 2016.

Variables	Categories	EIBF		COR	AOR
		No (%)	Yes (%)		
Maternal age (years)	15–24	168(26.8)	812(29.3)	1.31(1.03,1.63)	1.39(1.08,1.79)^a^
	25–34	291(46.3)	1413(51.0)	1.243(0.99,1.55)	1.16(0.92,1.45)
	≥35	169(26.9)	543(19.6)	1	1
Mother Currently working	No	461(73.4)	2153(77.8)	1.42(1.17,1.73)	1.45(1.19,1.78)^a^
	Yes	167(26.6)	615(22.2)	1	1
Wealth index	Poor	297(47.3)	1398(50.5)	1	1
	Medium	146(23.2)	662(23.9)	1.18(0.93,1.50)	1.19(0.93,1.54)
	Rich	185(29.5)	708(25.6)	1.19(0.95,1.49)	1.18(0.93, 1.51)
Number of under-five children	≤2	329(52.5)	920(33.2)	0.54(0.46,0.65)	0.52(0.43, 0.63)
	>2	298(47.5)	1847(66.8)		1
Place of delivery	Health facility	193(30.7)	861(31.1)	1.18(0.97,1.43)	1.25(1.01,1.53)^a^
	Home	435(69.3)	1907(68.9)	1	1
Mode of delivery	C/S	12(1.9)	14(0.5)		Reference
	Vaginal	616(98.1)	2753(99.5)	3.68(1.65,8.23)	4.06(1.75, 9.44)^a^
Sex of child	Male	328(52.2)	1294(46.8)	0.86(0.72,1.03)	0.84(0.69,1.01)
	Female	300(47.8)	1473(53.2)	1	Reference
Women participating in making health care decisions	Yes	431(68.6)	2136(77.2)	1.59(1.32,1.94)	1.52(1.25,1.85)^a^
	No	197(31.4)	632(22.8)	1	
Size of baby	Large	175(27.9)	831(30.0)	1.48(1.16,1.89)	1.49(1.17, 1.92)^a^
	Average	282(44.9)	1189(43.0)	1.13(0.92,1.39)	1.13(0.92, 1.39)
	Small	171(27.2)	748(27.0)	1	1

1: Reference, COR: crude odd ratio, AOR: Adjusted Odds Ratio, EIBF: Early initiation of breastfeeding CI: Confidence Interval.

a = p-value <0.05.

### 3.3 Factors associated with early initiation of breastfeeding among urban residences

In bivariate logistic regression analysis, variables such as educational status of the mothers, maternal working status, involvement of mother in making health care decisions, number of under-five children, wealth index, ANC, place of childbirth, mode of delivery, size of baby, birth order and SCC were associated with early initiation of breastfeeding at a p-value of <0.25 and adjusted in multivariable logistic regression to control the confounding factors. In multivariable logistic regression analysis, maternal participation in making health care decisions, SCC, mode of delivery, birth order, and ANC were identified as factors associated with early initiation of breastfeeding. Accordingly, the odds of early initiation of breastfeeding were 2.62 times (AOR: 2.62:95 % CI: 1.33, 5.17) higher among newborns whose mothers participated in making healthcare decisions than newborns whose mothers didn't participate in making healthcare decisions. The odds of early initiation of breastfeeding were higher among newborns whose mothers have 1–3 ANC (AOR: 5.76, 95 % CI: 1.91, 17.34) and ≥4 ANC (AOR: 3.64, 95 % CI 1.24, 10.67) than newborns whose mothers didn't have ANC. Newborns who delivered vaginally had 2.52 times higher (AOR: 2.52, 95 % CI: 1.15, 5.54) odds of early initiation of breastfeeding than newborns delivered through cesarean section. The likelihood of early initiation of breastfeeding was 1.94 times (AOR: 1.94, 95 % CI: 1.01, 3.75) higher among newborns whose birth orders are second to third than newborns whose birth orders are first. The odds of early initiation of breastfeeding were 2.58 higher (AOR: 2.58, 95 % CI: 1.44, 4.63) among newborns who received SCC than newborns who didn't receive SCC (Table 3).

**Table 3:** Shows the bivariate and multivariable logistics regression analysis of factors associated with early initiation of breastfeeding in urban residences, Ethiopia, 2016.

Variables	Categories	EIBF		COR(95%CI)	AOR(95%CI)
		No (%)	Yes		
**Maternal education status**	No formal education	22(25.9)	94(23.9)	1	1
	Primary	18(21.2)	133(33.8)	1.69(0.86,3.34)	1.58(0.71,3.54)
	Secondary and above	45(52.9)	166(42.2)	0.85(0.48,1.51)	0.62(0.28,1.35)
**Mother Currently working**	No	44(51.8)	217(55.4)	1.14(0.71,1.83)	0.89(0.51,1.57)
	Yes	41(48.2)	175(44.6)		1
**Women participating in making health care decisions**	Yes	68(79.1)	346(88.3)	1.95(1.06,3.58)	2.62(1.33,5.17)^a^
	No	18(20.9)	46(11.7)	1	1
**Number of under-five children**	≤2	64(75.3)	242(61.7)	0.53(0.31,0.89)	0.56(0.28,1.12)
	>2	21(24.7)	150(38.3)	1	1
**Wealth index**	Poor	9(10.6)	28(7.1)	1	1
	Medium	3(3.5)	1(0.3)	0.18(0.02,1.37)	0.26(0.02,3.54)
	Rich	73(85.90	363(92.6)	1.63(0.74,3.59)	2.51(0.86,7.35)
**Antenatal care visits**	Not all	13(15.3)	20 (5.1)	1	1
	1-3 visits	18 (21.2)	110 (28.1)	3.98 (1.69,9.34)	5.76 (1.91,17.34)^a^
	≥4 visits	54 (63.5)	262 (66.8)	3.19 (1.51,6.77)	3.64 (1.24,10.67)^a^
**Place of delivery**	Health facility	67 (78.8)	354 (90.1)	2.44 (1.36,4.53)	1.69 (0.68,4.24)
	Home	18 (21.2)	39 (9.9)	1	1
**Mode of delivery**	C/S	14 (16.5)	31 (7.9)	1	1
	vaginal	71 (83.5)	361 (92.1)	2.31 (1.17,4.55)	2.52 (1.15,5.54)^a^
**Size of baby**	Large	19 (22.4)	133 (33.8)	1.83 (0.93,3.60)	1.93 (0.90,4.14)
	Average	45 (52.9)	178 (45.3)	1.02 (0.57,1.82)	0.84 (0.44,1.63)
	Small	21 (24.7)	82 (20.9)	1	1
**Birth order**	1	43 (50.6)	119 (30.3)	1	1
	2–3	27 (31.8)	180 (45.8)	2.43 (1.42,4.14)	1.94 (1.01,3.75)^a^
	≥4	15 (17.6)	94 (23.9)	2.32 (1.21,4.44)	2.44 (0.93,6.35)
**Skin-to-skin contact care**	No	49 (57.0)	127 (32.4)	1	1
	Yes	37 (43.0)	265 (67.6)	2.76 (1.71,4.45)	2.58 (1.44,4.63)^a^

1: Reference, COR: crude odd ratio, AOR: Adjusted Odds Ratio, EIBF: Early initiation of breastfeeding CI: Confidence Interval.

a = p-value <0.05.

## 4 Discussion

This study has unveiled the variations of factors associated with early initiation of breastfeeding among rural versus urban residents. Accordingly, the age of mothers, maternal current working status, number of under-five children, and place of delivery and birth sizes are identified as factors associated with early initiation of breastfeeding among rural residences. Whereas, ANC, birth order, and SCC are the factors associated with early initiation of breastfeeding among urban residences. Moreover, participation of mothers in making health care decisions and mode of delivery are identified as factors associated with the early initiation of breastfeeding, regardless of the residence. The possible explanation for the disparities might be that mothers from urban residences are more likely to be educated, economically stable, and have access to health facilities as compared to mothers from rural residences [31].

In rural populations, newborns whose mothers are aged 15–24 years have higher odds of early initiation of breastfeeding than newborns whose mothers are aged ≥35 years. The finding is consistent with studies conducted elsewhere [32,33]. A plausible explanation could be the advancement of health education concerning the best practices for breastfeeding, education for girls, and the empowerment of women in making decisions about their health care [34].

In rural residences, this study revealed that newborns whose mothers are not currently working have higher odds of early initiation of breastfeeding than newborns whose mothers are currently working. The finding is consistent with the study done in Nigeria [35]. The possible explanation could be that mothers who are working can afford the fee for their baby's meal; this can result in formula feeding and delayed initiation of breastfeeding. Moreover, mothers who are working might be able to afford the cost of cesarean section as the mode of delivery, which is a known determinant of delayed initiation of breastfeeding [21].

In rural residences, the odds of early initiation of breastfeeding are higher among newborns who have large birth sizes than newborns who have small birth sizes. The finding is synonymous with the studies done in Nigeria and Nepal [35,36]. This could be that large-sized babies may be looked like healthy and the mothers and healthcare professionals may commerce breastfeeding within the first hours of life. Moreover, small-sized premature babies may require extra care immediately after birth to adjust their life outside the womb. Hence, the time intervals could increase the risk of delayed initiation of breastfeeding.

In rural residences, this study revealed that newborns who are delivered at health institutions have higher odds of early initiation of breastfeeding than newborns who are delivered at home. The finding is consistent with studies done elsewhere [14,35]. This is an expected finding that the healthcare professional at the health facility can assist the mother to start breastfeeding early in the first hours of birth.

In rural residences, our findings further indicate that the likelihood of early initiation of breastfeeding is lower among newborns whose mothers have two or fewer children in the household as compared to newborns whose mothers have two or more children in the household. The finding is inconsistent with the study done elsewhere [19]. This can be explained as that mothers could be informed about the benefit of optimal breastfeeding during their preceding births.

In the urban residence, the odds of early initiation breastfeeding are higher among newborns whose mothers have ANC than newborns whose mothers didn't have ANC. The finding is consistent with the studies conducted elsewhere [18,23,32,33,37]. This could be explained as ANC services may provide a platform for healthcare providers to educate expectant mothers about the benefit of early initiation of breastfeeding for newborns.

In urban residences, this study also found that newborns who received SCC from their mothers have higher odds of early initiation of breastfeeding than newborns who didn't receive SCC. The finding is synonymous with the study done elsewhere [38]. This is the fact that the sensory touch of mothers and newborns can increase oxytocin secretion, which is important to breast milk production [39].

In urban residences, this study found that newborns whose birth orders are second to third have higher odds of early initiation of breastfeeding as compared to newborns whose birth orders are first. The finding is supported by the study done in Nigeria [35]. This can be explained as mothers may change their behavior regarding the benefit of early initiation of breastfeeding from their previous experience.

Regardless of the residence, this study revealed that newborns who are delivered vaginally have higher odds of early initiation of breastfeeding than newborns who are delivered through cesarean section. The finding is synonymous with studies done elsewhere [38,[40], [41], [42]]. The possible justification might be that the pressure applied during surgical extraction or anesthesia provided during the procedure may reduce the activity of newborns, which can contribute to delayed breastfeeding initiation. Moreover, breastfeeding initiation following a cesarean section may be delayed due to the recovery period after the procedure and the time needed to repair the surgical incision.

The other finding regardless of residence, the likelihood of early initiation breastfeeding is higher among newborns whose mothers participated in making health care decisions than newborns whose mothers didn't participate in making health care decisions. The finding is inconsistent with the study done in Nigeria [35]. This could be that empowered mothers can refuse cultural norms or restrictions regarding the pre-lacteal feeding of infants [34].

Generally, the clinical and public health implications of this study are to increase the practices of optimal breastfeeding among infants, through to reduce neonatal mortality. Therefore, strategies and programs have to be endorsed against the aforementioned factors respective to the residence of mothers.

### 4.1 Strengths and limitations

The study can be broadly applied at the national level and utilized weighted data of study participants. We followed the rural versus urban data segregation method and disclosed the variation of factors associated with early initiation of breastfeeding among rural versus urban residences. However, the study was conducted using a cross-sectional study design, hence it is difficult to establish the cause-and-effect relationship. In this study, the sample size of study participants among rural versus urban residences was different despite data weighing being done to adjust the cluster effect. We used self-report data so, it is prone to recall biases, particularly for variables, time to initiation of breastfeeding, SCC practice, and birth size though an attempt has been taken to minimize recall bias.

## 5 Conclusions

This study concludes that the factors associated with early initiation of breastfeeding are different among rural versus urban residences. Accordingly, maternal ages, maternal current working status, birth sizes, and place of delivery are identified as factors associated with early initiation of breastfeeding among rural residences. Whereas, ANC, SCC, and birth order are identified as factors associated with early initiation of breastfeeding among urban residences. Regardless of residence, mode of delivery, and involvement of mothers in making health care decisions are common determinants of early initiation of breastfeeding. In rural residences, special emphasis and breastfeeding support should be provided for older mothers. Moreover, in rural residences, breastfeeding support should be provided to small size newborn babies to start breastfeeding early in the first hour. In urban residences, strengthening maternal and child health services utilization should be emphasized. Skin-to-skin contact care should be provided for all newborns at birth. Irrespective of the residence, special emphasis has to be given to newborns delivered by cesarean section to increase the rate of early initiation of breastfeeding. Furthermore, empowering women should be emphasized to improve early initiation of breastfeeding.

## Consent for publication

Not applicable.

## Funding

No funds were received for this study.

## Data availability statement

The data used for this study was deposited at the Harvard Dataverse Network repository:URL: https://doi.org/10.7910/DVN/XR7Z3M.

## CRediT authorship contribution statement

**Desalegn Girma:** Writing – original draft, Software, Methodology, Formal analysis, Data curation, Conceptualization. **Zinie Abita:** Writing – review & editing, Software, Methodology, Formal analysis, Data curation.

## Declaration of competing interest

The authors declare that they have no known competing financial interests or personal relationships that could have appeared to influence the work reported in this paper.

## Abbreviations

AOR: Adjusted odds ratio
CI: Confidence interval
DHS: Demographic Health Survey
EDHS: Ethiopian Demographic and Health Survey
SCC: skin-to-skin contact care
HSDP: Health Sector Development Program
WHO: World Health Organization
