# Supplementary material for: Rural versus urban variations of factors associated with early initiation of breastfeeding in Ethiopia
Source: Heliyon. 2024 Jun 23;10(13):e33427. doi: 10.1016/j.heliyon.2024.e33427 (PMC467058; doi:10.1016/j.heliyon.2024.e33427)
Supplement: Multimedia component 1 [file mmc1.docx]

**Supplementary Table 1**: Description and measurement of independent variables.

| **Variables** | **Categorization and description of variables** |
| --- | --- |
| Maternal age (years) | The age of mothers was originally categorized into seven categoriesas‘15–19’,‘20–24’,‘25–29’,‘30–34’,‘35–39’, ‘40–44’and‘45+’.For this study, the age of the mother was re-coded as ‘young’(15–24years),‘young adult’(25–34years) and‘ middle age’(35–49years) to get adequate samples for each category |
| Maternal education status | The original question ask the mother about the higher level of education do the mother was attended. The response options were ‘no education’, ‘primary education’,  ‘Secondary education ’and ‘higher education’. for this study we recoded into “No education”, primary education”, secondary and above” to get because few had reported having higher education. |
| Mother Currently working | The original question ask the respondent as “respondent currently working” and the response was “No and Yes”. |
| Partner education status | The original question ask the mother about the higher level of education do the mother was attended. The response options were ‘no education’, ‘primary education’,  ‘Secondary education ’and ‘higher education’. for this study we recoded into “No education”, primary education”, secondary and above” to get because few had reported having higher education |
| Number of under five children | The original question asked for the “number of children 5 and under in household”  And the response option was the number of under five children in the based on the mother’s in the house hold. For the analysis , we recoded as “ less than two child” and “greater or equal to 2 children” |
| Wealth index | Originally, the Wealth index was categorized as ‘very poor’, ‘poor’, ‘middle’, ‘rich’ and very rich. For this study , to obtain adequate samples in each category , “very poor” and “poor” was recoded as “poor” and “medium” recoded as “medium” and “rich” and “very rich” recoded as “rich” |
| Exposed to media | The media exposure status of mothers were measured using three variables; frequency of watching TV, reading a newspaper, and listening to the radio, and coded as “yes” if an individual was exposed to all or either of the three and “No” if an individual was not exposed to at least one of these. |
| Age at first birth |  |
| Place of delivery | The place where the mother gave the last birth and re-categorized as delivery at  Home and health facility |
| Women participating in making health care decisions | The women’s health care decision-making autonomy was assessed as the person who usually decides to obtain healthcare. Which was categorized as women participating in making health care decisions and didn’t participate in making health care decisions (decides by their husband/partner). |
| Antenatal care visits | The original question asked the mother as, ‘how many times did you receive antenatal care during the last pregnancy? And the response option was the number of antenatal care visits do the mother have .For this study, we recoded as “Not all”, having  “1-3 Antenatal care visits”, and greater or equal to four antenatal care visits. |
| Mode of delivery | The original question ask the respondent as delivery by caesarean section and the response were “no” and “Yes”. For this study we recoded as “ vaginal” and “caesarean section” |
| Parity | The original question ask the mother as “Entries in birth history” and the response was provide in number. For this study, we recoded, as “parity- one to three, four to five and greater or equal to six”. |
| Sex of child | Categorized as ‘male’ or‘ female’. |
| Birth type | The original question asked about the number of births for one pregnancy and the response were open ended, for this study, we recoded as ‘single birth ‘for one birth or  ‘Multiple birth’ for more than one birth. |
| Skin to skin care | The original question asked the mother as “Was child put on mother's bare skin after birth” and the response option was “No and Yes”. |
| Birth size | This is based on the maternal report about the size of the recent birth and it was recoded as “small, average and large” birth size. |
| Birth Order | The mother or caregiver was asked about the order of each children and the response one, two….etc. For this study we recoded as First-born , Second to three born and fourth or more |
| Age of children | The age of children was re categorized as <6month, 6-11 month and 12-23 month , to get enough sample size for each categories. |
